# Prevalence and associated factors of the psychological impact of COVID-19 among communities, health care workers and patients in Ethiopia: A systematic review

**DOI:** 10.1016/j.amsu.2021.102403

**Published:** 2021-05-25

**Authors:** Firomsa Bekele, Desalegn Feyissa Mechessa, Birbirsa Sefera

**Affiliations:** a Department of Pharmacy, College of Health Science, Mettu University, Mettu, Ethiopia; b School of Pharmacy, College of Medicine and Health Science, Mizan- Tepi University, Mizan-Aman, Ethiopia

**Keywords:** COVID-19, Associated factors, Prevalence, Psychological impacts, Ethiopia

## Abstract

**Introduction:**

The mental health effects of coronavirus is found to be high in health care professionals, patients and communities. Therefore, this review tried to summarize the prevalence and associated factors of the psychological impact of COVID-19 among the health care workers (HCWs), patients and communities in Ethiopia.

**Methods:**

The studies from Medline via PubMed, Science Direct, and Google Scholar were searched from February 17 to March 17, 2021. PRISMA-2020 (Preferred Reporting Items for Systematic Reviews and Meta-analyses) was used to conduct this review.

**Result:**

Initially, 2190 publications were obtained from three databases (PubMed, Science Direct, and Google Scholar). Finally, 9 articles that fulfilled eligible criteria were included in the review. Among different types of mental health impacts stress was reported that lies in the range from 18% to 100%, anxiety was reported from 27.7% to 100%, depression was from 12.4% to 55.7%. Several factors were associated with negative psychological impacts of COVID-19 among health care workers, patients and communities such as level of education, occupation, gender, age, marital status, presence of co-morbidity, lack of social support, personal/family exposure, their attitude, income level, family size, presence of respiratory symptoms, substance use, area of residence, and lack of protective equipment.

**Conclusion:**

There was overall high psychological impact of COVID-19 pandemic among healthcare workers, communities, and patients. The most common indicators of psychological impact reported across studies were anxiety and stress. Therefore, online psychotherapy and cognitive behavioral and mindfulness-based therapies should be provided through smartphone applications to minimize psychological impacts of COVID-19.

## Highlights

• Coronavirus disease (COVID-19) is a new pathogen of virus that was first discovered in Wuhan.
• The emergency of this coronavirus has resulted in a variety of mental health impact.
• The psychological impact of coronavirus is found to be high in health care professionals and the community.

## 1 Introduction

Coronavirus disease (COVID-19) is a new pathogen of virus that was first discovered in Wuhan, China, and which is currently infects human unlike SARS, MERS, and influenza virus [1,2].

The emergency of this coronavirus has resulted in a variety of mental health impact such as major depressive disorder, fear, and stress [3]. The increase in negative psychological impacts of this disease is as the result of the rapid spread of the virus, increased access to information and higher case fatality rate [4,5].

During this pandemic period, having mental and psychological problems leads to poor self-care practice, appetite, sleep, immunity status, and compliance to the instructions given by health care provider that exposed them to infectious etiology [6].

The psychological impact of coronavirus is found to be high in health care professionals and the community [1,7]. Among different types of psychological impacts, anxiety, depression, panic attacks, or psychotic symptoms were highly reported [8,9].

The mental health impact of a disease outbreak is usually neglected during pandemic management although the consequences are costly. Early evidence has shown that health workers directly involved in the diagnosis, treatment, and care of patients with COVID-19 are at risk of developing mental health symptoms [10].

Different risk factors can predispose to the high burden of the mental health impacts such as being a female, increase in age, the presence of concomitant disease, inadequate social support, increased the number of family size, low socio-economic status, occupations and level of educations [1,3,6]. Besides this the fear of being contagious and infecting others, physical exhaustion, and a lack of sufficient protective equipment are a predictors of poor mental health impacts [[11], [12], [13]].

Know days the COVID-19 pandemic became widely affects the community, patients and health care providers in Ethiopia [11]. The psychological effects of this disease might be high in developing country including Ethiopia as the result of lack of resources, unorganized health care system, and inadequate health care professionals. Despite a scanty of reviews were conducted on the mental health impacts of COVID-19 globally, there was no systematic reviews done in Ethiopia and the culture and socio-demographic characteristics of the included participants were different which necessitates the need to summarizes the variety of findings among community, patients and HCWs in Ethiopia.

## 2 Methods

### 2.1 Searching strategy

The objective of the review was to conclude the magnitude and risk factors of poor mental health effects of COVID-19 in Ethiopia. The protocol of PRISMA-2020 was used to undertake this systematic review [14]. Three authors namely FB, DFM, and BS were involved in searching different literatures from three data bases like PubMed, Science Direct, and Google Scholar. The time period used to conduct this review was from the February 17 to March 17, 2021. The searched literatures were imported into Endnote X5 to eliminate any duplication. The MESH term for the database was (Psychological impacts AND Coronaviruses disease-19 AND Healthcare workers AND Community AND Patients OR determinants OR prevalence AND Ethiopia).

### 2.2 Eligibility criteria

The published articles on magnitude and associated factors of COVID-19 in Ethiopia having a primary outcome and full texts available were included. The articles with unknown primary outcomes, systematic reviews and meta-analysis studies, not peer reviewed and commentary to editors were not eligible.

### 2.3 Data abstraction

The articles fulfilling the inclusion criteria were extracted on separate data sheet. The outcome of the interest extracted were the socio-demographic characteristics, different psychological impacts along with the authors name and year of the study.

### 2.4 Methodology quality assessment

To assess the quality of the methodology the National Institutes of Health (NIH) Quality Assessment tool was used [15]. As per the tool 5 researches were good [11,[16], [17], [18], [19]],3 were fair [1,6,20], 1 article was poor [21].

## 3 Results

### 3.1 Search results

**Fig. 1:**
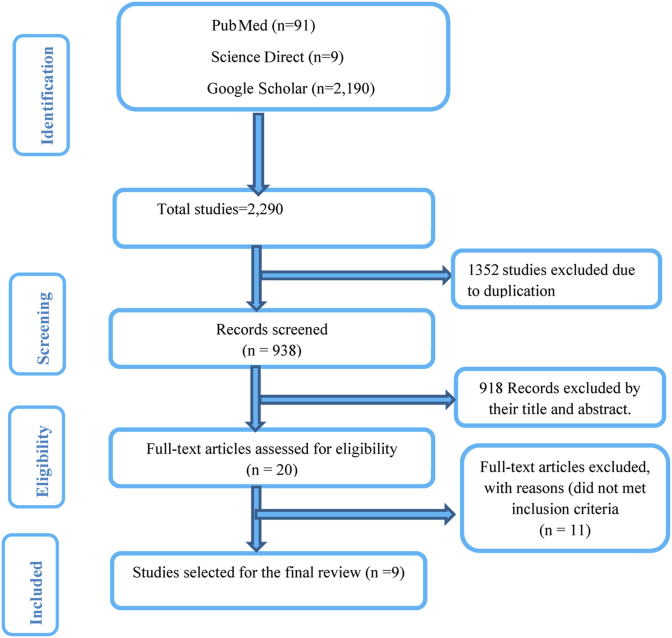
Flow chart of the systematic research and study selection process.

A total of 2190 articles were obtained up on initial searching from PubMed, Science Direct, and Google Scholar. A total of 1352 articles were removed due to duplications. Finally a total of 918 articles were excluded by observing their title and abstracts. Consequently, only 20 articles were subject to a full-text review. Finally, 9 articles were selected to be included in our review (Fig. 1).

### 3.2 Characteristics of studies included in this review

In our review the filtered articles were cross-sectional studies. Articles included in this study were conducted among patients, health care workers and community. The majority of the participant were male in five of the articles [1,11,16,17,21], whereas female was predominant in the three articles [6,18,19] and one articles was only done among female students [20]. Regarding to the study participants three of articles were conducted on health care workers [1,11,18],two of them were on community [6,21]. Another two articles were on patients, one articles was on university students [16], whereas one articles was on women attending perinatal services [20] (Table 1).

**Table 1:** Summary of baseline characteristics of the articles that were previously published and included studies in the systematic review, 2021.

Primary author	Year of publication	Study design	(study setting/population)	Average age in years	Sample size	Gender (Female %)	Occupation/Educational status
**Kassaw CH et al** [6]	2020	Cross-sectional	Community	–	420	55%	• Unemployed 22.8 = %) • Housewife = 14.2% • Student = 22.62% • Private work = 21.1% • Government employee = 19.0%
**Aylie NS et al** [16]	2020	Cross-sectional	Students	22.58 ± 2.8	322	36.6%	• Students = 100%
**Teshome A et al** [11]	2020	Cross-sectional	Health Care Workers	29.29 ± 5.69	798	39.6%	• Nurse = 44.6% • Doctor = 8.1% • Medical laboratory = 10.5% • Midwifery = 15.0% • Pharmacist = 9.6% • Public health officer = 12.0%
**Chekole YA et al** [1]	2020	Cross-sectional	Health Care Workers	• 18–24 = 19.7% • 25–31 = 66.4% • >31 = 13.9%	244	34%	• Doctor = 11.1% • Nurse = 41.0% • Health officer = 6.1% • Midwifery = 9.8% • Laboratory technology = 9.8% • Pharmacist = 9.4% Others = 12.7
**Kassaw CH et al** [20]	2020	Cross-sectional	Women's attending at the perinatal service	• 28 ± 5.6	178	100%	• Primary = 53.3% • Secondary and above = 46.6%
**Adhena G et al** [21].	2020	Cross-sectional	Community	• 38.6 ± 12	422	48.3%	• Government = 49.8% • Merchant = 25.8% • Others = 24.4%
**Hajure M et al** [17]		Cross-sectional	Chronic Medical Patients	• 43.3 ± 13.3	411	64.8%	• Government worker = 24.1% • Self-employed = 30.7% • Un-employed = 45.3%
**Kibret S et al** [18]		Cross-sectional	Healthcare workers	• 20–29 = 48.9% • 30–39 = 39.7% ≥40 = 11.5%	305	65.9%	• Diploma = 12.8% • BSc Degree = 73.8% • MSc and above = 13.4%
**Addis SG et al** [19]		Cross-sectional	Chronic disease patients	48.2 ± 15.83	413	52.1%	• Housewives = 38.5% • Employed = 20.6% • Students = 14.8% • Farmer = 14.5% • Unemployed = 5.1% • Merchant = 6.5%

### 3.3 Risk of bias

The blindness and hiddenness of the outcomes were sufficient in 5 articles [1,6,16,18,19] and not known in remaining 4 articles. The study populations was unknown in 3 articles and well known in the remaining 6 [6], [16], [1], [17], [18], [19][1,6,[16], [17], [18], [19]]. We have obtained complete outcome variables in all articles except one article [6]. Other types of bias were not found in our systematic review.

### 3.4 Prevalence of the psychological impact of COVID-19

Anxiety was the common psychological impacts in the study of Kassaw CH et al. 36% [6].and Hajure M et al. 61.8% [17], whereas three articles were only measures anxiety [11,18,20].Stress was only predominant according to study of Aylie NS et al. 32.5% [16],whereas the study of Chekole YA et al. and Adhena G et al. solely reported stress as psychological impacts of COVID-9 [1,21]. Among different psychological impacts of COVID-19 depression was the least commonly occurred in three findings of Kassaw CH et al. 12.4% [6], Aylie NS et al. 21.2% [16] and Hajure M et al. 55.7% [17] [Table 2].

**Table 2:** Summary of included studies on Prevalence and Associated Factors of Psychological impact of COVID-19 among Community, Health Care workers and Patients in Ethiopia, 2021.

Author		Psychological impact	Determinants
**Kassaw CH et al** [6]		• Anxiety = 36% • Depression = 12.4% • Stress = 18%	• Gender, • Educational status, • Monthly income, • Family size, • Contact with the person came abroad • History of chills and fever
**Aylie NS et al** [16]		• Depression = 21.2% • Anxiety = 27.7% • Stress = 32.5%	• Being female, • Staying at home, • History of medical illness • Poor and moderate social support • Not living with their parents, • Relatives got coronavirus • Low family income • Substance use • Previous psychological co-morbidity
**Teshome A et al** [11]		• Anxiety = 100%	• Contact with confirmed or suspected cases • No COVID-19 updates • No confidence on coping with stresses • COVID-19-related worry • Their feelings
**Chekole YA et al** [1]		• Stress = 100%	• Being at the age range of 25–31 years • Level of education • Being Nurse • Being pharmacist
**Kassaw CH et al** [20]		• Anxiety = 100%	• Living in Rural area • Primary level of education • Poor social support • Primigravida
**Adhena G et al** [21].		• Stress = 100%	• Being illiterate • Having a chronic disease • Being merchant • Not implementing preventive measures • Not following policies and scientific evidence to COVID-19
**Hajure M et al** [17]		• Depression = 55.7% • Anxiety = 61.8%	• Female gender • Poor social support • Marital status • Longer duration of illness • Presence of co-morbidity • Age • Tobacco use
**Kibret S et al** [18]		• Anxiety = 100%	• Marital status • Age • Having chronic illness • Having suspected COVID-19 family members • Not having an access to Personnel Protective Equipments
**Addis SG et al** [19]		• Minimal (Normal) psychological impact = 77.2% • Mild psychological impact = 15.0% • Moderate psychological impact = 5.6% • Severe psychological impact = 2.2%	• Age • Longer duration of illness • Being female • Presence of respiratory symptoms • Lack of social support

### 3.5 Factors associated with the psychological impact of COVID-19

It was found that variable factors were associated with increase in psychological impacts of COVID-19. In our review being a female was the most predictors of psychological impacts of coronavirus disease according to the reports of Kassaw CH et al., Aylie NS et al. Hajure M et al. and Addis SG et al. [6,16,17,19]. Similarly age was a determinants of mental health effects of COVID-19 in four reports of Chekole YA et al., Hajure M et al., Kibret S et al. and Addis SG et al. [1,[17], [18], [19]]. The level of educations was strongly associated with the increased risk of different psychological effects like anxiety, depression and stress in three findings of Kassaw CH et al., Chekole YA et al. and Kassaw CH et al. [1,6,20].

Apart from the socio-demographic factors lack of social support during coronavirus pandemics was a determinants of negative mental health impacts according to the study of Aylie NS et al., Kassaw CH et al., Hajure M et al. and Addis SG et al. [16,17,19,20].

Substance use was a predictors of anxiety, depression and stress in two findings of Aylie NS et al. and Hajure M et al. [16,17].Families having COVID-19 was the most risk factors for developing the negative mental health effects in two studies of Aylie NS et al. and Kibret S et al. [16,18] [Table 2].

## 4 Discussion

The results of our study revealed a high prevalence of anxiety and stress among patients, community and health care workers during the outbreak of COVID-19 virus. This is consistent to the finding of China [3],Oman [22]. However, anxiety and depression was the most commonly occurred psychological impacts according to the study of Luo M et al., 2020 [9].The study of Nepal and China showed that anxiety, depression and insomnia were the most commonly occurred mental health impacts [10,23]. In Saudi Arabia depression was the widely occurred psychological features [24]. The variable reports of psychological impacts might be due to the fact that different socio-demographic characteristics and psychological comorbidities of included HCW, patients and community across different country.

In this study, female and young participants were more likely to experience moderate to severe anxiety, stress and depression compared to males [1,6,[16], [17], [18], [19]]. Similar findings have been reported in Oman [22]. The study of Luo M et al., 2020 also founds high rates of psychological impacts among womens [9]. On the contrary, gender was not a predictors of anxiety and stress in Peru [25]. This is due to women may be more stressed during lockdown, as they may be over proportionately burdened by childcare duties.

It was found that Social support correlated with less mental health problems [16,17,19,20]. This is consistent with the study of Muller AE et al., 2020 [12]. Similarly, perceived social support was negatively correlated to Depression, anxiety and stress scale scores in China [3].

Inadequate Protective measures and contact history were the independent risk factors for Psychological features [6,11,16,18,21]. This is inline to the study of In China [26]. However, contact history was not have determined the mental health impacts in Indian community [27].

The presence of social drug use like tobacco have a the association with the psychological effects of COVID-19 [16,17].This is differ to the finding of Indonesia [28].This is due to the increase in risk of psychological features as the results of substance use that can precipitates the mental health impacts of COVID-19.

As a limitation, all included studies were cross-sectional, which was difficult to identify causal effect relationships. The other weakness includes the limited number of published articles was obtained, meta-analysis was not performed and heterogeneity of the articles, all psychological impacts were not studied in some articles. Besides this, a search for the gray literature was not conducted and only published, peer-reviewed articles were included and most articles failed to report proper randomization techniques.

## 5 Conclusion

In the current systematic review, we have observed an overall high psychological impact of COVID-19 pandemic among healthcare workers, community, and patients. The most common indicators of psychological impact reported across studies were anxiety and stress. Common risk factors of heavier psychological burden included being women, younger age, poor social support, substance use, occupation, marital status, educational status, monthly income, and contact history. Therefore, online psychotherapy and cognitive behavioral and mindfulness-based therapies should be provided through smartphone applications to minimize a various determinants of psychological impacts of COVID-19. Besides to this HCWs should strongly provide mental health support to their clients by waiving fees for mental health services, offering additional support and increasing awareness of mental health.

## Availability of data and materials

The datasets used are available from the corresponding author on a reasonable request.

## Consent

Not applicable. No individual person's personal details, images or videos are being used in this study.

## Ethical approval

Not applicable.

## Funding

No funding was received.

## Author contribution

FB involved in the searching of the literature, extraction of results and editing the manuscript. DFM involved in the interpretation and revision of this systematic literature review. BS involved in searching of the literature and editing the manuscript.

## Registration of research studies

1. Name of the registry: RESEARCH REGISTRY, https://www.researchregistry.com.

2. Unique Identifying number or registration ID: reviewregistry1130.

3. Hyperlink to the registration (must be publicly accessible): https://www.researchregistry.com/register-now#home/registrationdetails/5d70f2520791fb0011b79e9f/

## Guarantor

The Guarantor is the one or more people who accept full responsibility for the work and/or the conduct of the study, had access to the data, and controlled the decision to publish.

Firomsa Bekele.

## Declaration of competing interest

The authors declared that they have no competing interest.

## Abbreviations

COVID: Coronavirus disease
HCW: Health care workers
NIH: National Institute of health
PRISMA: Preferred Reporting Items for Systematic Reviews and Meta-analyses
UK: United Kingdom
USA: United States of America
